# Three-Dimensional Cell and Tissue Patterning in a Strained Fibrin Gel System

**DOI:** 10.1371/journal.pone.0001211

**Published:** 2007-11-21

**Authors:** Takuya Matsumoto, Jun-Ichi Sasaki, Eben Alsberg, Hiroshi Egusa, Hirofumi Yatani, Taiji Sohmura

**Affiliations:** 1 Department of Oromaxillofacial Regeneration, Osaka University, Suita, Japan; 2 Department of Biomedical Engineering, Case Western Reserve University, Cleveland, Ohio, United States of America; Center for Genomic Regulation, Spain

## Abstract

Techniques developed for the *in vitro* reproduction of three-dimensional (3D) biomimetic tissue will be valuable for investigating changes in cell function in tissues and for fabricating cell/matrix composites for applications in tissue engineering techniques. In this study, we show that the simple application of a continuous strain to a fibrin gel facilitates the development of fibril alignment and bundle-like structures in the fibrin gel in the direction of the applied strain. Myoblasts cultured in this gel also exhibited well-aligned cell patterning in a direction parallel to the direction of the strain. Interestingly, the direction of cell proliferation was identical to that of cell alignment. Finally, the oriented cells formed linear groups that were aligned parallel to the direction of the strain and replicated the native skeletal muscle cell patterning. In addition, vein endothelial cells formed a linear, aligned vessel-like structure in this system. Thus, the system enables the *in vitro* reproduction of 3D aligned cell sets replicating biological tissue patterns.

## Introduction

Biological tissues have specific cell patterns and well-organized structures that can be observed microscopically (e.g., highly oriented myofibers in skeletal muscle and the hexagonal structure of the liver lobule comprised of hepatocytes). Techniques that reproduce these 3D cell and tissue patterns *in vitro* would be helpful for the development of advanced tissue engineering–based therapies. Progress has been made in the study of patterning with regard to cells and extracellular matrix proteins [1]–[3] in two-dimensional (2D) cell culture systems. For example, Whitesides et al. introduced a technique that manipulates protein patterning using soft lithography and succeeded in controlling 2D cell patterning on the matrices [1]. However, few techniques are available for the regulation of cell and tissue patterning in three-dimensional (3D) cell culture systems [4], [5]. The 3D cell patterning in hydrogel systems has been established using a 3D printing system for cell seeding [6], however, the cell patterning created by this technique may rapidly become disordered as a result of cell migration and proliferation. Thus, the most complex issue regarding 3D cell patterning is achieving control of the direction/position of cell proliferation and migration during the cell culture period. In this study, we hypothesized that the structural control of 3D cell culture matrices would guide the 3D cell patterning which we desire to organize.

Fibrin, which is formed by the mixture of fibrinogen and thrombin purified from peripheral blood, is found in wound healing regions. Not only is it used therapeutically as surgical glue but fibrin has also been investigated for its use as a biocompatible and biodegradable material in biomedical engineering applications (e.g., drug delivery systems [7]–[9], tissue engineering [10]–[12]). Fibrin gels, which are comprised of hydrophilic cross-linked fibrils, are considered suitable for 3D cell culture.

To obtain matrices that can reproduce highly oriented 3D cell patterning similar to that of skeletal muscle tissue or 3D vasculature, we attempted to control the micro-architecture of fibrin matrices using mechanical tensile force. We subsequently evaluated cultured myoblasts and vein endothelial cells in the strained fibrin gel.

## Results

### Strained fibrin gel

**Figure 1:**
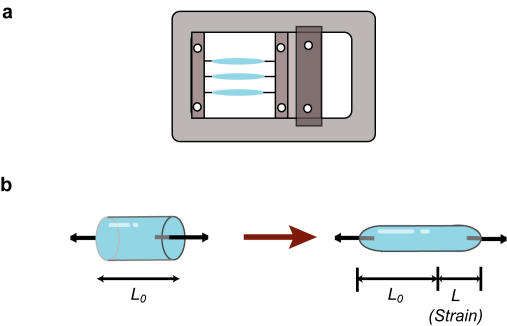
Application of strain to fibrin gels. a) Fibrin gels were subjected to continuous tensile strain that was applied using a custom-made device. b) The length of gel extension (*L*) from the initial gel length (*L_0_*) was defined as the applied strain (*L/L_0_•100*%).

**Figure 2:**
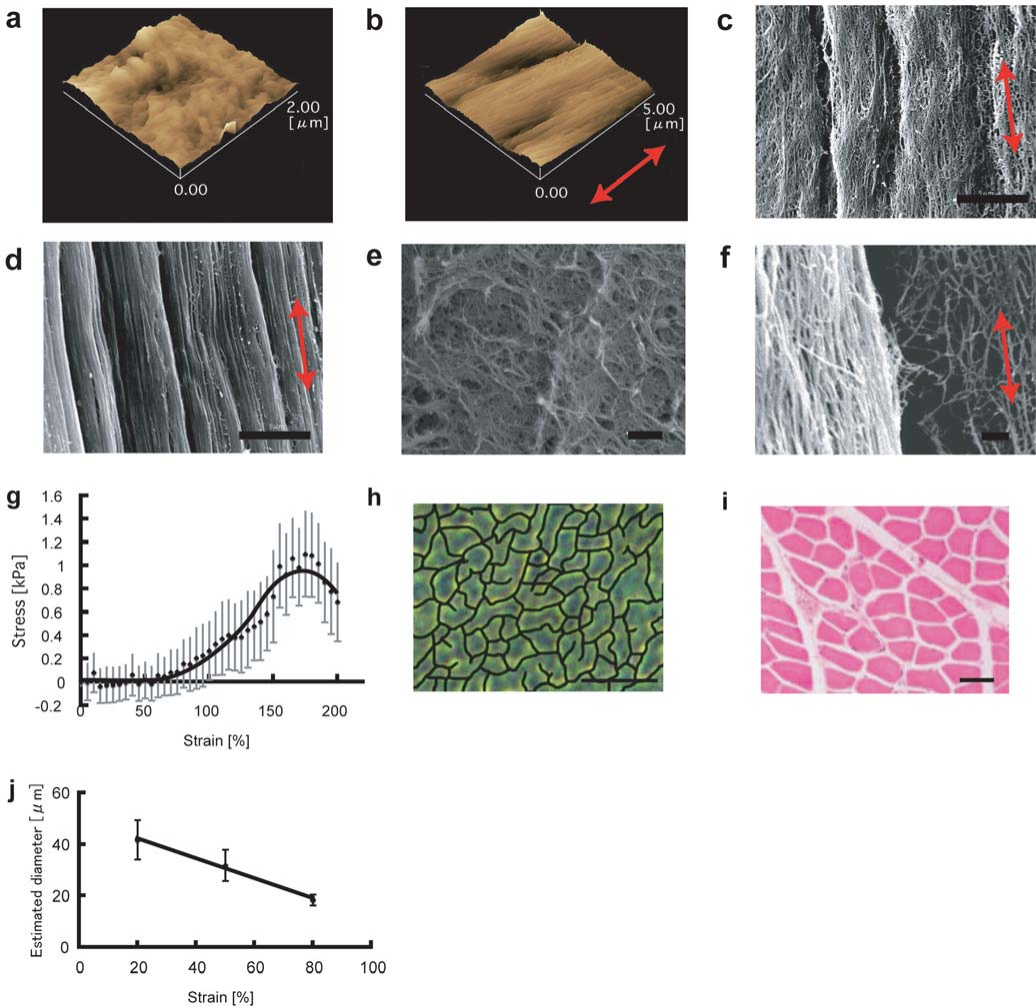
Orientation of fibrin fibrils and formation of bundle-like structures in fibrin gels. a, b) AFM images of a control fibrin gel (a) and a strained fibrin gel (b, 50% strain). c, d) SEM images of bundle-like structures formed in a strained fibrin gel (c, 25% strain; d, 100% strain; bar: 5 µm). e) SEM image of a control fibrin gel (bar: 1 µm). f) SEM image of the border of the bundle-like structure in a strained fibrin gel (bar: 1 µm). g) Typical strain-stress curve of a fibrin gel used in this study. h) Representative image of a cross-section of a strained fibrin gel as observed under a phase-contrast microscope (TE2000, Nikon, Japan; bar, 100 µm). The border of the bundle-like structure was highlighted using the brush tool of Adobe Photoshop software (Adobe, CA, USA). i) A cross-section of native rat skeletal muscle tissue (Hematoxylin-eosin staining, HE). Each bundle exhibits a polygonal shape and the morphology resembles that in the cross-section of the strained fibrin gel shown in (h) j) The cross-sectional area of individual bundle-like structures was measured using image-analysis software (Lumina Vision, Mitani, Japan) and extrapolated to circular cross-sections to calculate the average diameter of the bundles under strains of specific magnitudes. The red arrow in the figure indicates the strain direction.

We used surgical sutures fabricated from poly (lactic-co-glycolic acid) (PLGA) to tether the fibrin gels and to apply tensile strain to them. Each fibrin gel was formed in a cylindrical silicone mold (length, 10 mm; diameter, 6 mm) with sutures inserted at both ends. The sutures were then clamped to a custom-made device that was used to generate continuous tensile strain (0% to 200%) (**Fig. 1a,b**). The suture material possessed a highly rough texture due to its woven structure, and it firmly attached to the fibrin matrices. Atomic force microscopy (AFM) images revealed that the fibrils were randomly folded in the fibrin gel prior to strain application and subsequently they extended in the strain direction due to the tensile strain (**Fig. 2a,b**). Scanning electron microscopy (SEM) images indicated that bundle-like structures were formed in the strained gels; this structure comprised fibrin fibrils that were oriented parallel to the strain direction (**Fig. 2c,d**). The control fibrin gel exhibited a mesh-like structure wherein the fibrils did not show any specific orientation (**Fig. 2e**). Highly magnified SEM images indicated that the fibril alignment in the above structure depended on the amount of strain applied (**Fig. 2c,d**).

The fibrils were torn at the border of each bundle (**Fig. 2f**); this suggested that the displacement of each set of fibrils to the strain direction due to the tensile strain facilitated the formation of bundle-like structures in the fibrin gel. Interestingly, the strain-stress curve for this gel revealed two distinct stages. No internal force was detected in the hydrogel until approximately 50% strain, and thereafter, the force gradually increased up to 1.1 kPa (**Fig. 2g**). These results indicated that the fibrin fibrils were likely undergoing alignment and the formation of bundle-like structures during the initial tensile strain up to 50%. The cross-section of the bundle-like structures generated exhibited a polygonal shape, not a complete circular shape (**Fig. 2h**), which is similar to the cross-section of natural skeletal muscle tissue (**Fig. 2i**). The estimated diameter of each bundle-like structure decreased with increasing strain application (**Fig. 2j**).

### 3D aligned cells and cell sets cultured in the strained fibrin gel

**Figure 3:**
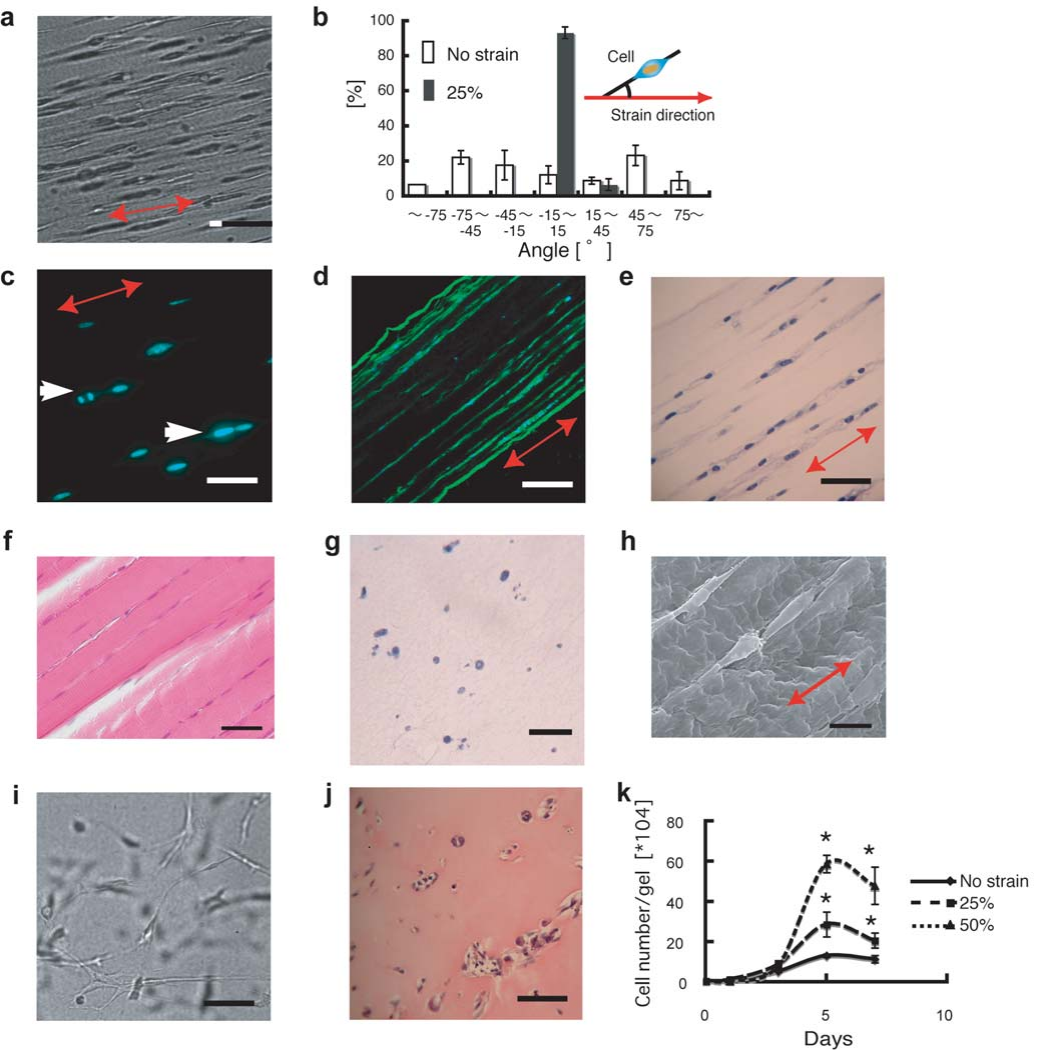
Myoblasts in strained fibrin gels. a) Myoblasts patterning in a strained fibrin gel (Bar: 100 µm). b) The alignment of randomly selected cells was determined by using image-analysis software (Bar: 200 µm). c) Nuclear staining of myoblasts in a strained fibrin gel. The arrows indicate proliferating cells (Bar: 100 µm). d) Linearly aligned cell groups formed in a strained hydrogel (Bar: 400 µm). e) Hematoxylin-eosin (HE) staining of linearly aligned myoblasts sets(Bar: 200 µm). f) HE staining of a longitudinal section of rat skeletal muscle tissue (Bar: 50 µm). g) A cross-section of a strained fibrin gel containing myoblasts. The cells were evenly distributed in the hydrogel, and adjacent cells were not in contact with each other (HE staining, bar: 50 µm). h, i) SEM image of myoblast positions in a strained fibrin gel (h) (Bar: 100 µm) and in a control fibrin gel (i) (Bar: 100 µm). j) HE staining of myoblasts in a control fibrin gel (Bar: 200 µm). k) Cell proliferation in the fibrin gels subjected to different strains. Asterisks indicate significant difference (p<0.01). The red arrow in the figure indicates the strain direction.

Next, we investigated cell dynamics within this fibrin gel system. The fibrinogen solution used to form the fibrin gel included 2.5×10^4^ myoblasts/ml (C2C12), and the gel was continuously subjected to 25% strain. The cells in the fibrin gel displayed a specific alignment that was parallel to the strain direction (**Fig. 3a, b**). Interestingly, the direction of cell proliferation was identical to that of cell alignment (**Fig. 3c**). Therefore, one seeded cell divided multiple times, and the oriented cells subsequently formed linear groups that were aligned parallel to the strain direction (**Fig. 3d,e**), which is similar to the cellular organization found in a longitudinal section of native skeletal muscle tissue (**Fig. 3f**). Cross-sections of the gel revealed that adjacent cell groups were not in contact with each other (**Fig. 3g**). These results suggest that the positions of the cells in the fibrin gel are restricted to the spaces between the fibrin bundles such that they align and proliferate parallel to the strain direction. This assumption is supported by a typical SEM image (**Fig. 3h**) that showed cells positioned in the spaces between the bundle-like structures of the fibrin gel. In contrast, the cells in the non-strained fibrin gel (control gel floating in medium solution) displayed spread cell morphologies with random orientations, and they were linked to each other in various directions (**Fig. 3i,j**).

Cell proliferation in the hydrogel at different levels of strain was then investigated. The results indicated that cell proliferation varied depending on the magnitude of applied strain. Cell proliferation in the strained gel conditions was significantly higher than that in the control gel. Moreover, compared to the gels subjected to lower strain (25%), the gels subjected to higher strain (50%) demonstrated enhanced cell proliferation (**Fig. 3k**). This functional variation in cell proliferation was probably caused by compressive stress and matrix stiffness differences between the gels that in turn depended on the strain applied [13]–[16].

### 3D aligned vessel in the strained fibrin gel

**Figure 4:**
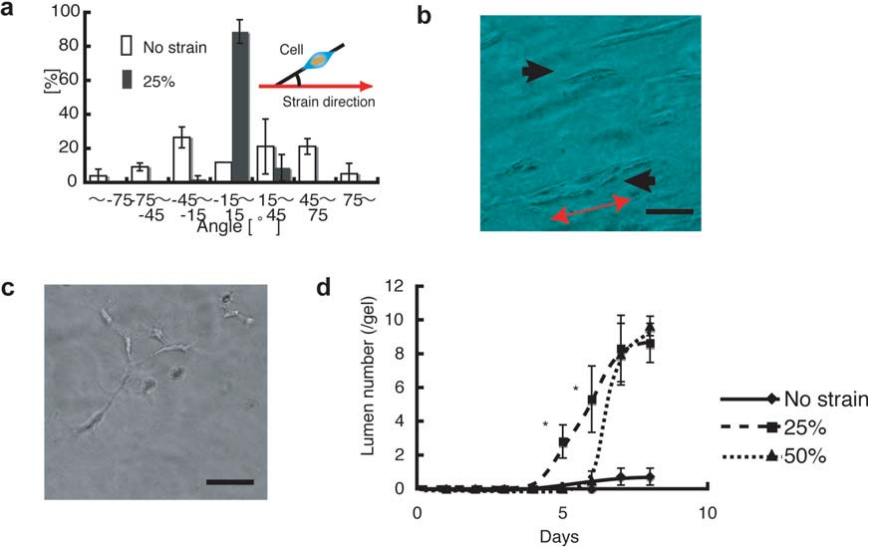
HUVECs in strained fibrin gels. a) Alignment of HUVECs in a strained fibrin gel. b) Aligned vessel-like structure in a fibrin gel (arrows). c) HUVECs in a control fibrin gel. d) Cells that developed lumens in a fibrin gel system. Asterisks indicate significant difference (p<0.01). The red arrow in the figure indicates the strain direction. The scale bars are 100 µm for b and c.

To investigate the ability of this oriented hydrogel to guide the alignment and growth of different types of cells, we cultured human umbilical cord vein endothelial cells (HUVECs) in the fibrin gel system and applied a range of different strains. Initially, the HUVECs aligned and proliferated in a specific direction that was parallel to the strain direction, similar to that observed for the myoblasts culture (**Fig. 4a**). However, the cell morphology changed over time, and the cells began to form lumens after 5 days of culture. Unbranched vessel-like structures that were aligned in the direction of applied strain formed during the 8 day culture period, while the cells in the control gel exhibited random orientation (**Fig. 4b,c**). Vessel formation was observed under each strain condition; the cells in the gel condition subjected to a higher strain demonstrated delayed vessel formation compared to those in the gel condition subjected to a lower strain (**Fig. 4d**).

## Discussion

In previous studies, hydrogel orientation was achieved using magnetic fields [17]–[19] and tensile strain [20]. Although individual cells were reported to align well in a specific direction using these hydrogel systems, they did not proliferate to form linearly aligned cell groups. The mesh-like fibril structure formed in hydrogels (e.g. collagen gel) used in previous studies may have disturbed cell proliferation in a specific direction [17], [18]. In this study, the formation of bundle-like structures in the hydrogel is likely a key factor for the formation of linearly aligned cell sets, and our unique gel-loading methods resulted in this anisotropic structure. Previous studies have also used cyclic mechanical strain [21]–[23] or electrical stimulation [24] to achieve cell patterning and muscle-like tissue formation. Although it is assumed that the specific cell alignment observed in these studies was caused by the external stimuli, our results suggest that structural changes in the gel provided contact guidance cues to promote cell alignment. As illustrated, our system facilitates the reproduction of aligned cell and tissue patterning such as that observed in skeletal muscle, blood vessels, and neurons by the simple application of continuous strain to the fibrin gel. Our study also indicates that cell proliferation and differentiation might be controlled by the applied strain. This biomimetic tissue reproduced *in vitro* would allow one to understand the cellular behaviors in tissues [25] without conducting animal experiments, and would be valuable in the development of novel tissue engineering-based therapies.

Although our system permits the reproduction of 3D aligned cell and tissue patterning that is observed *in vivo*, controlling the maturation of fabricated tissue (e.g., regulation of fibrin degradation and extracellular matrix formation by cells) is also crucial to obtain functional *in vitro* engineered tissues. Previous reports indicated that cyclic strain application to cells promoted matrix production from cells and enhanced the mechanical properties of 3D cell and matrix constructs [26], [27]. Hence, the application of different types of strain to 3D patterned cells in our system may be helpful in regulating the maturation of engineered tissues.

## Materials and Methods

### Fibrin gel preparation

Fibrinogen solution (3 mg/ml; Sigma, MO, USA) containing aprotinin (5 mg/ml, Sigma) was mixed with thrombin solution (2 U/ml, Sigma) in a 1∶1 ratio. Subsequently, 400 µl of this solution was poured into a silicone mould (length, 10 mm; diameter, 6 mm) and incubated (37°C) for 20 min. For culture in the fibrin gel, cells (2.5×10^4^ cells/ml) were added to the fibrinogen solution prior to the gelation. In order to apply continuous strain to the hydrogel, surgical PLGA sutures (Φ = 0.25 mm, Alfresa, Japan) were inserted into the solution in the silicone mould at both the ends prior to the gelation. The formed gels were removed from the mould, and the sutures were then clamped at various distances by using a custom-made device.

### Cell culture

Myoblasts (C2C12, Riken cell bank, Japan) were cultured in Dulbeco's Modified Eagle's Medium (DMEM) containing 10% fetal bovine serum (FBS). In the proliferation analysis, DMEM containing 10% FBS was used for initial 4 days, and then DMEM containing 2% horse serum was used for the rest of the culture period. Human umbilical cord vein endothelial cells (HUVECs, cc-2517, Cambrex, MD, USA) were cultured in Endothelial Growth Media -2 (EGM-2, Cambrex) medium containing 2% FBS. To count the cells in the fibrin gel, the gel was dissolved in trypsin/EDTA solution at 37°C for 30 min, and the detached cells were directly measured using a hemocytometer.

### Cell alignment analysis

For individual cell alignment analysis, a minimum of 30 cells in the fibrin gel were randomly selected in phase contrast images (TE2000, Nikon, Japan), and the long axis angle of selected cells (relative to direction of strain application = 0°) was measured using image analysis software (Lumina vision, Mitani, Japan).

### SEM and AFM observation

Fibrin gels were fixed with 4% paraformaldehyde and dried by critical point drying (HCP-2, Hitachi, Japan). AFM (SPM9500, Shimadzu, Japan) was performed in the tapping mode. Samples sputtered with gold were used for SEM observation (JSM6390, JEOL, Japan).

### Section staining

Fibrin gels were fixed with 4% paraformaldehyde and embedded in paraffin. Thin paraffin sections (4 µm) were stained with hematoxylin and eosin or with anti-skeletal myosin (raised in rabbit, Sigma) followed by a goat anti-rabbit secondary antibody labeled with Alexa Fluor488 (Invitrogen, CA, USA). Immunofluorescent nuclear staining was carried out by using Hoechst 33342 (Invitrogen). Phase contrast images of cross-sections of the bundle-like structures in the fibrin gels were adjusted for brightness and contrast, and the borders of each bundle-like structure were highlighted by using the brush tool in Photoshop 7.0 software (Adobe, CA, USA). Fluorescent microscopy (TE2000, Nikon, Japan) images were captured using a CCD camera (Coolsnap cf, Photometrix, AZ, USA).

### Statistics

Statistical analysis of data was accomplished by one factor analysis of variance (ANOVA). Student t test was used for comparison at a 99% confidence interval. Data points and error bars in the graphs represent the mean and SD calculated from four independent experiments.
